# Small bowel-small bowel intussusception with high grade obstruction due to intramural submucosal ileal hamartoma in a 5-year-old child: A case report

**DOI:** 10.1016/j.ijscr.2019.05.044

**Published:** 2019-05-31

**Authors:** Federico Scorletti, Kevin Bove, Rebeccah L. Brown

**Affiliations:** a Division of Pediatric General and Thoracic Surgery, Cincinnati Children’s Hospital Medical Center, 3333 Burnet Avenue, Cincinnati, 45229, OH, USA; b Neonatal Surgical Unit, Department of Medical and Surgical Neonatology, Bambino Gesù Children’s Hospital, IRCCS, Piazzale Sant’Onofrio, Rome, 00164, Italy; c Department of Pathology and Laboratory Medicine, Cincinnati Children’s Hospital Medical Center, 3333 Burnet Avenue, Cincinnati, 45229, OH, USA

## Abstract

**Introduction:**

Intussusception is a common cause of emergency in children. We report a unusual case of intestinal obstruction due to small bowel-small bowel intussusception with an intestinal hamartoma as the lead point.

**Presentation of the case:**

A 5 year old boy presented to the ED with worsening abdominal pain, emesis and bloody diarrhea. An abdominal radiograph showed evidence of small bowel obstruction and US confirmed a small bowel-small bowel intussusception with a likely cystic mass as the lead point. Given these findings, surgery was performed and revealed an intestinal hamartoma as a lead point.

**Discussion:**

Intussusception in children is mostly idiopathic and usually amenable to reduction by air enema. The presence of a lead point is unusual and usually requires surgical intervention.

**Conclusion:**

We report an unusual case of intussusception with small bowel obstruction due to intestinal hamartoma, a rare pathologic finding as a lead point for intussusception. Ultrasound was useful for diagnosing the intussusception and confirming the presence of a lead point.

## Highlights

• Intussusception in children can present with evidence of small bowel obstruction.
• Ultrasound can show a pathologic lead point which requires surgical intervention.
• Surgery should begin with a laparoscopic approach and converted to open procedure if needed.

## 1 Introduction

Intussusception is the most common cause of abdominal emergency in infants and young children often presenting with signs of intestinal obstruction [1]. Most cases are classified as “idiopathic”, and the incidence of intussusception secondary to a lead point such as Meckel’s diverticulum, polyp, duplication cyst, or tumor is only about 5–10% [2]. The majority of cases of childhood intussusception are ileocolic, idiopathic [3] and reducible by air enema in 85–90% of cases. Clinically significant small bowel-small bowel intussusceptions are rare in children, more commonly associated with a lead point, and more likely to require surgical intervention. We present an unusual case of high-grade intestinal obstruction due to small bowel-small bowel intussusception with an intestinal hamartoma as the lead point.

## 2 Presentation of the case

**Fig. 1:**
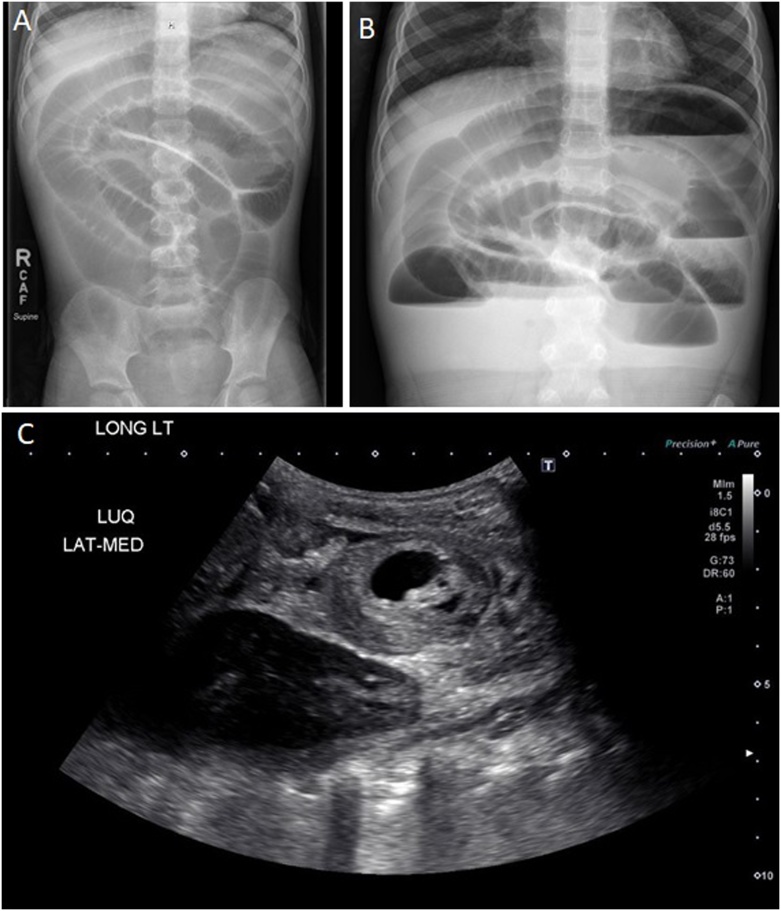
Preoperative imaging. Panel A: antero-posterior abdominal X-ray in decubitus position Panel B: antero-posterior abdominal X-ray in standing position; Panel C: abdominal ultrasound with the arrow pointing at the intussusception.

**Fig. 2:**
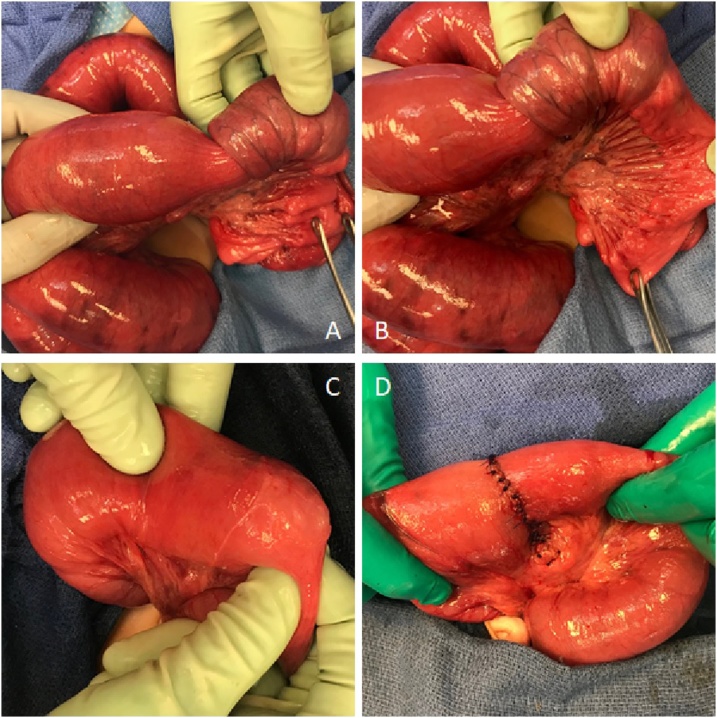
Picture from surgery. Panel A and B: manual reduction of small bowel-small bowel intussusception; panel C: cystic structure arising from the ileal wall; panel d: end-to-end ileo-ileal anastomosis after segmental resection.

A 5  year old boy with no significant past medical or surgical history presented to the ED with 4 day history of worsening non-bilious emesis and crampy left-sided abdominal pain, with a few episodes of diarrhea and a small amount of blood in the stool. On physical exam, he was afebrile with normal vital signs. He appeared quite fussy, and his abdomen was markedly distended, tympanic, and moderately tender without appreciable masses or peritoneal signs. No peritoneal signs were present. On auscultation, high pitched bowel sounds were heard. Laboratory analyses were normal. An abdominal radiograph (Fig. 1, panels A and B) showed numerous dilated loops of small bowel with multiple air-fluid levels on the upright view consistent with a high-grade small bowel obstruction. The clinical history, exam, and radiologic findings raised concern for small bowel obstruction with a high index of suspicion for intussusception given his age and no history of previous surgery. An ultrasound (Fig. 1, panels C and D) confirmed a small bowel-small bowel intussusception in the left abdomen extending over a length of approximately 7 cm with a likely pathologic lead point described as a cystic structure measuring 3 cm, possibly an enteric duplication cyst or necrotic polyp. Given these findings, the patient was taken to the OR for exploratory laparoscopy, however, upon insertion of the scope, marked distension of the bowel loops precluded adequate visualization, and we felt it was unsafe to proceed. The initial umbilical incision was then extended longitudinally, converting the procedure to a mini-laparotomy. A small bowel-small bowel intussusception of about 20 cm was found in the left abdomen, easily reduced with gentle manipulation. The bowel appeared pink and well perfused without signs of ischemic injury. A cystic structure arising from the ileal wall was found as lead point. We resected the segment of small bowel including the lead point lesion and then performed a end-to-end ileo-ileal anastomosis (Fig. 2). The patient had an uneventful post-operative course, tolerating a regular diet by POD 4, and was discharged on POD 5.

**Fig. 3:**
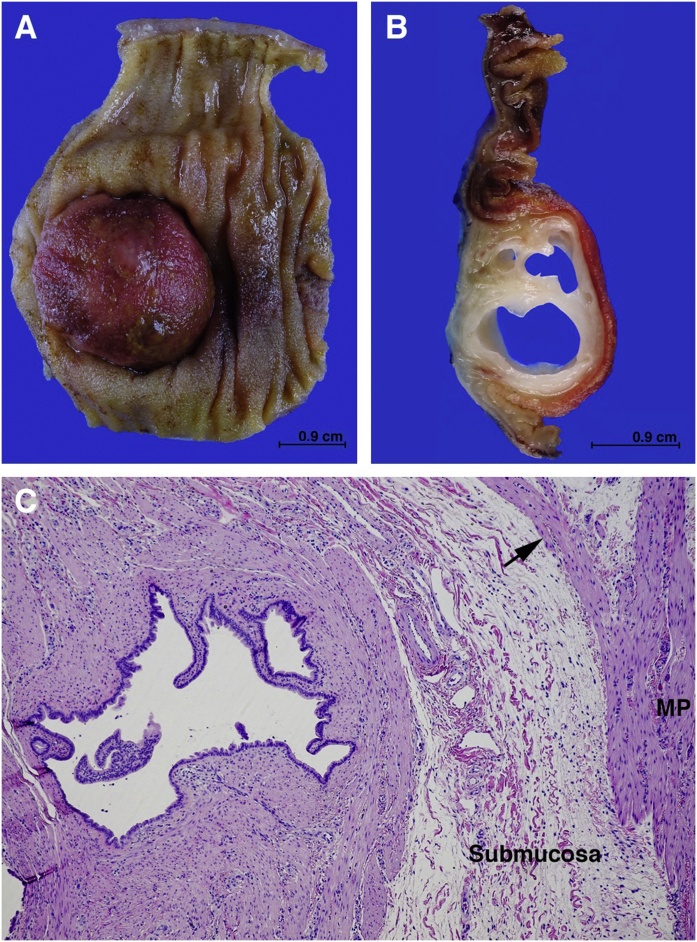
Pictures of the pathology specimen. Panel A: raised mucosal nodule; panel B: submucosal multi-loculated cyst on the cut surface; panel C: multi-cystic submucosal lesion.

Pathology revealed a 5.2 cm long segment of small intestine with a 2.8 × 2.5 × 1.1 cm raised mucosal nodule (Fig. 3, Panel a). The mucosa overlying the nodule was granular red-brown and sectioning revealed a submucosal multi-loculated cyst on the cut surface (Fig. 3, panel b). The cysts were filled with a thin clear mucinous material. Microscopy showed a complex circumscribed but not encapsulated multi-cystic submucosal lesion with localized deficiency of the muscularis propria. (Fig. 3, panel c). The cysts of various sizes were lined by intestinal epithelium with occasionally brush border (small intestinal epithelium) and focally was more colonic in character. No Paneth cells are identified. The components of a cystic lesion were enclosed within disorganized bundles of mature smooth muscle focally contiguous with the deficient layers of the muscularis propria. Contiguous with the cystic lesion were supernumerary vascular channels of varying size, most of which were dilated and only a few of which had smooth muscle walls consistent with malformed veins. Most of the vasculature component appeared to be distended, possibly malformed lymphatics. Final diagnosis was intramural submucosal hamartoma composed of intestinal cysts, smooth muscle, and malformed veins and lymphatics.

## 3 Discussion

Intussusception is one of the most common causes of intestinal obstruction in infancy, secondary only to pyloric stenosis, with an incidence of 56 children/100,000/year 1].

More than 90% of the case are idiopathic and 10% are secondary to a pathologic lead point such as Meckel’s diverticulum, polyp, cyst or lymphoma [4]. Two-thirds of these can be identified on ultrasound [5] and patients tend to be older than 2 years [6]. Ileocolic intussusception is the most common form and usually is treated with an air enema. Relatively common is the incidental finding of transient small bowel-small bowel intussusception on ultrasound. These usually reduce spontaneously without any treatment, are not associated with a pathologic lead point, and rarely lead to intestinal obstruction. In contrast, a small group of children may present with persistent small bowel-small bowel intussusception complicated by obstruction [7] and clinical deterioration [8]. These can present with a wide spectrum of clinical features without specificity. The classical presentation of intussusception (colicky abdominal pain, vomiting, and currant jelly stool) is present in only 25% of the cases [9]. Small bowel-small bowel intussusceptions are more likely to be associated with a lead point, and while the intussusception can be readily diagnosed by ultrasound, characterization of the actual lead point can be more difficult before surgery. The US finding of a long length of small bowel intussusception associated with high grade small bowel obstruction and free intraperitoneal fluid should raise suspicion for a pathologic lead point [10]. Furthermore, because of the more proximal nature of small bowel-small bowel intussusceptions, air enema reduction is unlikely to be successful, and repeated attempts may only delay definitive treatment and lead to bowel necrosis and perforation. Small bowel hamartomas are an exceedingly rare in both children and adults but have been associated with intussusception in a few case reports. In a review of the literature, Ikegami et al. found only 20 cases of small bowel hamartoma, 12 of which presented in pediatric population, 10 in the ileum, almost all causing intussusception [11]. Lin et al. [6] reported only one case of intestinal hamartoma as a cause of ileocolic intussusception in a series of 65 children with intussusception due to pathologic lead points, the most common of which was Meckel diverticulum.

Our patient was a previously healthy boy who presented with progressive symptoms of intestinal obstruction. Given his age and no prior history of surgery, we were suspicious for intussusception as the etiology of his symptoms. Ultrasound confirmation of a small bowel-small bowel intussusception with a pathologic lead point led us to perform a diagnostic laparoscopy without any attempt at reduction with an air enema.

Laparoscopic surgery is a safe and effective approach for the treatment of intussusception. A systematic review by Apelt et al. [12] of 276 cases of attempted laparoscopic reduction of intussusception revealed a 71% laparoscopic success rate. Unfortunately, in our case, the degree of bowel distension prevented optimal visualization, and conversion to open mini-laparotomy was necessary. The intestinal hamartoma was relatively large and contiguous with the intestinal wall precluding wedge resection and necessitating resection of the segment of bowel containing the lesion to avoid the risk of stenosis. The adjacent bowel appeared healthy, pink and well perfused, allowing a safe primary anastomosis with minimal effect on bowel length.

Intussusception secondary to a lead point can present with non-specific signs and tends to recur if the lead point is not identified. Clinically significant small bowel-small bowel intussusceptions are rare in children and usually associated with a pathologic lead point. Diagnosis can usually be made with ultrasound, air enema is not indicated, and urgent surgical intervention is warranted.

## 4 Conclusion

We report an unusual case of small bowel-small bowel intussusception with high grade small bowel obstruction due to intestinal hamartoma, a rare pathologic finding as a lead point for intussusception. Ultrasound was useful for diagnosing the intussusception and the presence of a lead point, although the unusual diagnosis was identified later on pathology of the resected specimen. This case report has been written in concordance with the SCARE criteria [13].

## Conflicts of interest

All the authors have no financial disclosures.

## Sources of funding

This research did not receive any specific grant from funding agencies in the public, commercial, or not-for-profit sectors.

## Ethical approval

This article does not contain any personal information that can lead to the identification of the patient.

Our institution allows for exempt case reports as long as less than 5 participant in the case.

## Consent

Written informed consent was obtained from the patient’s guardian for publication of this case report and accompanying images. A copy of the written consent is available for review by the Editor-in-Chief of this journal on request.

## Author contribution

Federico Scorletti:

Paper design, data collection and interpretation, paper writing, picture preparation.

Kevin Bove:

Data collection, writing the paper, picture preparation.

Rebeccah Brown:

Paper design, paper review.

## Registration of research studies

N/A.

## Guarantor

Federico Scorletti, Rebeccah Brown.

## Provenance and peer review

Not commissioned, externally peer-reviewed

## References

[1] Parashar U.D., Holman R.C., Cummings K.C., Staggs N.W., Curns A.T., Zimmerman C.M. (2000). Trends in intussusception-associated hospitalizations and deaths among US infants. Pediatrics.

[2] Lehnert T., Sorge I., Till H., Rolle U. (2009). Intussusception in children--clinical presentation, diagnosis and management. Int. J. Colorectal Dis..

[3] Rubenstein J.C., Liu L., Caty M.G., Christison-Legay E.R. (2015). Pathologic lead point is uncommon in ileo-colic intussusception regardless of age. J. Pediatr. Surg..

[4] DiFiore J.W. (1999). Intussusception. Semin. Pediatr. Surg..

[5] Miller S.F., Landes A.B., Dautenhahn L.W., Pereira J.K., Connolly B.L., Babyn P.S. (1995). Intussusception: ability of fluoroscopic images obtained during air enemas to depict lead points and other abnormalities. Radiology.

[6] Lin X.K., Xia Q.Z., Huang X.Z., Han Y.J., He G.R., Zheng N. (2017). Clinical characteristics of intussusception secondary to pathologic lead points in children: a single-center experience with 65 cases. Pediatr. Surg. Int..

[7] Fiegel H., Gfroerer S., Rolle U. (2016). Systematic review shows that pathologic lead points are important and frequent in intussusception and are not limited to infants. Acta Paediatr..

[8] Ko S.F., Tiao M.M., Hsieh C.S., Huang F.C., Huang C.C., Ng S.H. (2010). Pediatric small bowel intussusception disease: feasibility of screening for surgery with early computed tomographic evaluation. Surgery.

[9] Daneman A., Navarro O. (2003). Intussusception part 1: a review of diagnostic approaches. Pediatr. Radiol..

[10] Zhang Y., Dong Q., Li S., Ren W., Shi B., Bai Y. (2016). Clinical and ultrasonographic features of secondary intussusception in children. Eur. Radiol..

[11] Ikegami R., Watanabe Y., Tainaka T. (2006). Myoepithelial hamartoma causing small-bowel intussusception: a case report and literature review. Pediatr. Surg. Int..

[12] Apelt N., Featherstone N., Giulani S. (2013). Laparoscopic treatment of intussusception in children: a systemic review. J. Pediatr. Surg..

[13] Agha R.A., Borrelli M.R., Farwana R., Koshy K., Fowler A., Orgill D.P., For the SCARE Group (2018). The SCARE 2018 statement: updating consensus Surgical CAse REport (SCARE) guidelines. Int. J. Surg..

